# The Influence of Moisture Absorption and Desorption by the ABS Filament on the Properties of Additively Manufactured Parts Using the Fused Deposition Modeling Method

**DOI:** 10.3390/ma17091988

**Published:** 2024-04-25

**Authors:** Adam Hamrol, Błażej Góralski, Radosław Wichniarek

**Affiliations:** Faculty of Mechanical Engineering, Poznan University of Technology, Piotrowo 3, 60-138 Poznan, Polandradoslaw.wichniarek@put.poznan.pl (R.W.)

**Keywords:** additive manufacturing, fused deposition modelling, ABS, filament moisture, moisture absorption, moisture desorption

## Abstract

This paper presents the results of research on the influence of the moisture content in a filament made of ABS polymer on the properties of products manufactured using FDM (fused deposition modeling). Tests were carried out on a standard printer, using the parameters recommended by the manufacturer and the literature on the subject. A special climatic chamber was used to condition the material. A negative impact of ABS filament moisture on the strength and dimensional accuracy of printed products and on the structure of their surface is demonstrated. When the range of the filament moisture is between 0.17% and 0.75%, the strength decreases by 25% and the sample thickness increases by 10%. It is also shown that this effect does not depend on the history of the polymer reaching a given moisture level, i.e., by absorbing moisture in the absorption process or releasing moisture in the desorption process.

## 1. Introduction

Among additive technology methods, the most commonly used is FDM (fused deposition modeling) [1,2,3]. The process, commonly referred to as fused filament fabrication (FFF), is classified as material extrusion (MEX) according to ISO/ASTM 52900 [4]. In this manufacturing process, the modeling material is a thermoplastic capable of changing under the influence of heat from a solid state to a semi-liquid state, and vice versa. The modeling material is most often used in the form of a thin fiber, 1–3 mm in diameter, called a filament. The possibility of modeling is the result of melting the filament supplied from the extruder to the printing head. It is heated in the head to a temperature appropriate for a given filament, usually in the range of 190–280 °C, and then distributed in a semi-liquid form through the head nozzle, layer by layer, in accordance with the geometry contained in the CAD file, which is converted into printer code by a dedicated program, the so-called Slicer [5]. The result is a finished product, in practice also called a 3D print.

The materials used in the FDM process are polymers, which are materials consisting of macromolecules of organic compounds with an amorphous structure. The most commonly used materials are acrylonitrile-butadiene-styrene (ABS), polylactide (PLA), polyamide (PA, nylon), glycol-modified polyethylene terephthalate (PETG), polycarbonate (PC), polyoxymethylene (POM), polyethylene (PE), polyvinyl alcohol (PVA), and, in particular, a number of chemically and thermally stable materials such as polypropylene (PP), polyetheretherketone (PEEK), polyetherimide (PEI), and polyphenylsulfone (PPSU) [6]. Most of these materials are not indifferent to environmental conditions, including ambient humidity. Therefore, it is important to understand the impact of these conditions on the course and results of the modeling process, and, above all, on the properties of products manufactured [7,8,9].

The ingress of water molecules from the environment into the polymer is the result of the absorption of moisture from the environment and its diffusion within the material. Diffusion involves the transportation of molecules through chaotic collisions of molecules of a diffusing substance among themselves and/or with molecules of the surrounding medium, resulting in a reduction in chemical potential differences and a state of equilibrium. The rate of diffusion is proportional to the moisture concentration gradient. This process is described by Fick’s laws of diffusion [10,11].

Absorption involves the movement of water molecules from the environment to the internal structure of the material [12]. This process takes place at the phase boundary. The degree of moisture absorption depends on the type of material (absorption properties of the material depend on the available free volume between the chains) and environmental conditions, such as temperature and humidity [13]. The opposite of absorption is desorption [7,13]. The moisture release rate during desorption depends on the strength of the moisture bond with the polymer. If the bond strength is high, a higher temperature or longer time is needed to desorb. Moisture absorption causes changes in the bonding forces in the polymer and causes the phenomenon of the softening and swelling of plastic. Excessive water content in polymers can generate water vapor during additive processing, leading to the formation of defects such as voids and bubbles in prints. This can cause changes in the internal structure and the reduced strength of products made of polymer [14].

Water is absorbed by most polymers, but a change in properties is induced only in certain types of polymers. Due to their susceptibility to absorbing water, hygroscopic and non-hygroscopic polymers are distinguished. In non-hygroscopic materials, moisture settles only on their outer surface. However, in hygroscopic materials, water molecules are absorbed deep into the material. The materials used in FDM include hygroscopic polymers, such as ABS and PA. Non-hygroscopic polymers include PLA and PP (polypropylene) [14]. Haidi’s work showed [15] that in the case of a filament made of ABS, moisture absorption reaches a certain maximum level of 1.1% after 30 days of storage in a humid environment. This is also confirmed by research carried out by Kariz [16] in which, for ambient humidity RH = 87%, a humidity level of approximately 1.1% was achieved after 16 days. In the case of other polymeric materials, this maximum level is 1.3% for PLA after 16 days [16], 0.8% for PEI after 1 day (25 h) [17], 0.5% for PET-G, and 0.7% for PLA after 7 days [18].

The material’s hygroscopicity is dependent on its additives [19]. Banjo’s research showed, for example, that the addition of wood fibers to the PLA fiber causes a significant increase in hygroscopicity. Filaments with 10% additional fibers stored in a humid environment were characterized by approximately three times higher moisture content compared to fibers without additives stored in the same conditions. The addition of fibers in an amount of approximately 30% resulted in an almost fivefold increase in moisture content. Osman and Aita tested the addition of rice grass to the PLA polymer. As the volumetric share of rice grass increased from 5% to 30%, the filament moisture changed from 2% to 4% under the same ambient conditions [20].

In the above-mentioned work by Haidi [15], it was indicated that the moisture contained in the ABS filament affects its viscosity and fluidity, and increases the diameter of the filament material. This change in flow conditions indirectly affects the properties of prints but does not block the extruder nozzle. In the works [21,22,23,24], the tensile strength, elongation, and creep phenomenon of samples made of ABS, PLA, PC, nylon, and fiber composites with different moisture content were tested. All these studies showed a negative effect of moisture on the above-mentioned mechanical properties.

The research conducted by Hamrol [8] has shown that as the content of moisture in the ABS filament increases, the tensile strength of the printed samples decreases. A change in the content of filament moisture by 10 percentage points causes a decrease in the tensile strength of the sample by approximately 0.54 MPa, and that increased content of moisture has a negative impact on the surface quality of printed samples. With a low moisture content in the material, less surface roughness and more visible filament paths were obtained than in the case of samples printed from filaments with high water content.

The negative impact of ambient temperature and moisture on the properties of FDM printed parts is also confirmed by research results published in [25,26]. In these studies, the tendency of the filament material to creep was tested. The study used a climatic chamber allowing for the maintenance of the temperature in the range of 30–60 °C and air humidity in the range of 0–90%. It has been shown that high humidity and storage temperature of the filament can cause permanent deformation of products made of ABS. Valerga, in turn, showed that the moisture contained in the filament material affects the degradation of the material and combined with the high temperature of the printer nozzle, causes bubbles to form in 3D prints.

The relationship between environmental conditions and the geometry and mechanical properties of bisphenol polycarbonate FDM printed parts was investigated by Fang [27]. Samples with low moisture content were characterized by lower porosity and higher shape and dimensional accuracy than samples made from materials with high moisture content. Tests also showed that high porosity caused by moisture absorption significantly degrades the mechanical properties of printed samples.

Work is also being carried out on the possibility of compensating for the negative impact of moisture on the mechanical properties of samples produced using FDM, e.g., by selecting appropriate printing parameters. In [28], it was shown that the greatest interaction with the filament moisture is the thickness of the applied material layer. The higher the water content in the filament, the smaller the layer thickness should be in order to obtain appropriate print properties.

In research carried out by the team of Kim, the influence of storage conditions of ABS samples on their mechanical properties was determined. The parameters tested were humidity and ambient temperature. It has been shown that increased ambient temperature increases the rate of water absorption by printed prints but does not affect the amount of moisture absorbed [24].

In summary, it can be stated that the susceptibility of polymers to absorb moisture and its impact on the properties of products printed using FDM has been the subject of many studies. The results of this work clearly indicate this impact is definitely negative. It reduces the strength properties of prints and the shape reproduction of manufactured products [21,29,30]. However, there is a lack of studies on the impact of the way the filament achieves a given moisture level, i.e., by absorption or desorption, on the product properties. It can be assumed that the structure of a filament with the same water content, but obtained as a result of absorption or desorption, is different, which may in turn affect the properties of the manufactured products.

It should be noted that the issue of the presence of water in the processed polymer applies not only to additive manufacturing, but also, for example, to injection molding. In the latter case, the water content in the material is subject to even greater restrictions than in the case of the FDM method, because the water evaporating in the injection process has a very limited ability to escape from the mold.

The authors of the present study primarily aim to fill a research gap in the form of determining the effect of the way the ABS filament has gained certain water content on the strength properties and dimensional accuracy of the products manufactured from such filament, using FDM.

## 2. Research

### 2.1. Goals and Methodology

The aim of the research was to learn if the way the filament material obtains a certain moisture content, i.e., by absorbing or desorbing moisture, affects the strength of the effect of the moisture on the properties of products printed from it.

The research was carried out on ABS filaments because they are one of the most popular materials used in 3D printers. Acrylonitrile provides products made of ABS with high chemical resistance and hardness. Butadiene gives the material elasticity because it absorbs impact energy. Styrene, on the other hand, has a positive effect on thermal and processing properties, such as plasticity. Thanks to the individual components, ABS is characterized by high mechanical strength. The material has a melting point of 230 °C and is commonly used in industry due to its properties and potential for mechanical processing. It is used, among others, as a material for device casings, for the production of devices, spare parts and many others.

The product made of the ABS filament was in the form of a standard sample used in strength tests.

The following hypotheses were put forward:

- H0—the properties of products printed using FDM depend on the filament moisture but do not depend on the way the filament achieves a specific moisture level.
- H1—the properties of products printed using FDM depend on the filament moisture and the method of obtaining a specific moisture level by the filament.

The research was carried out in three stages:

Stage 1. Preparation of two batches of ABS material (filament):

- Dry filament (DF)—moisture content approximately 0.2%;
- Wet filament (MF)—moisture content approximately 0.75%.

Stage 2. Storing prepared batches of filament in a climatic chamber in the environment:

- Dry environment (DE)—humidity in the chamber 30%, chamber temperature 18 °C;
- Moist environment (ME)—humidity in the chamber 80%, chamber temperature 30 °C;

and:

- Testing moisture content of each batch of filament after 0, 1, 2, 4 and 7 days of storage;
- Preparing samples from the filament after 1, 2, 4, and 7 days of storing the filament. On each day, 5 samples were manufactured;
- Measurement or assessment of the properties of printed samples.

The hypotheses were verified by comparing the properties of samples made of filaments subjected to moisture absorption or desorption. The properties that were measured or assessed are as follows:

- Tensile strength of samples;
- Thickness and width of samples;
- Mass of samples;
- Surface structure of samples.

Stage 3. Storing two batches of samples printed from dry filament (DF) and wet filament (MF) in a climatic chamber in a moist environment (ME); the humidity in the chamber was 80%, the chamber temperature was 30 °C, and then we tested the water content of each batch of prints after 0, 1, 2, 4, and 7 days of storage.

**Figure 1:**
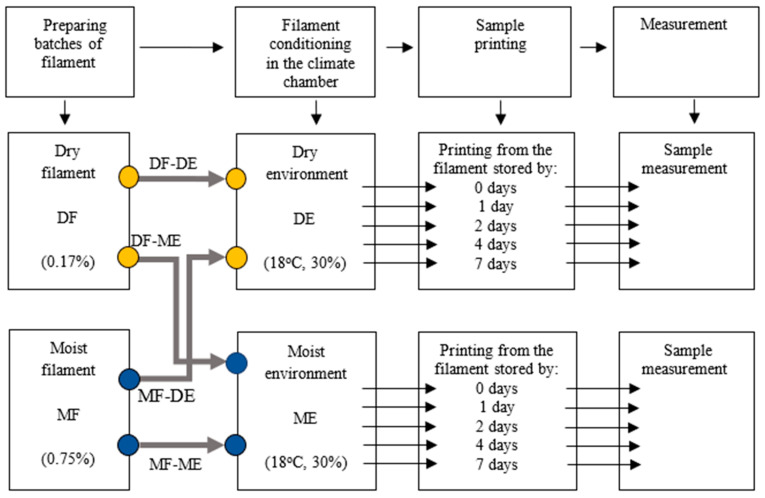
Diagram illustrating the sequence of research.

A diagram illustrating the idea and sequence of research in stages 1 and 2 is presented in Figure 1.

### 2.2. Research Stands

Specialized equipment was used in the research, including the following:

- Climatic chamber;
- A moisture analyzer for determining the moisture content of the filament;
- A 3D printer with a closed chamber;
- Strength testing machine;
- Digital microscope.

**Figure 2:**
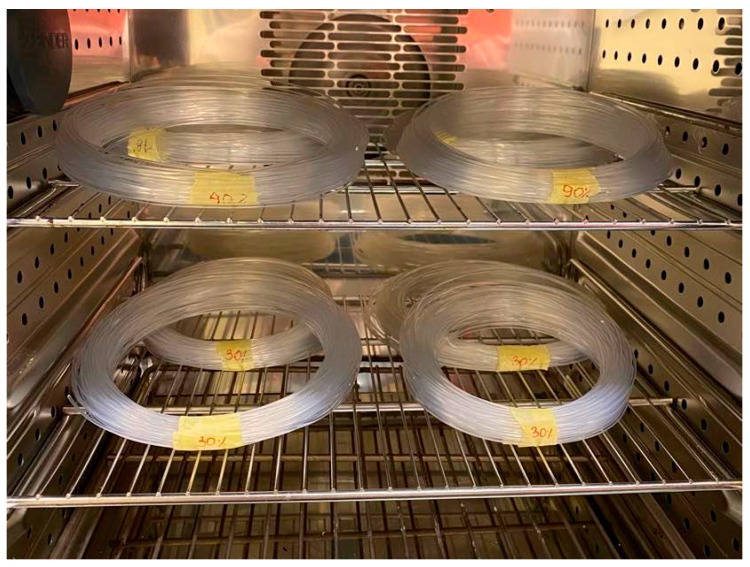
Arrangement of filament coils in the climatic chamber.

The BINDER MKF climatic chamber (E5) was used to condition the filament and the printed samples [Figure 2]. Samples were taken from a spool of a brand new, pure, transparent ABS filament manufactured by Fiberlogy (Poland).

**Figure 3:**
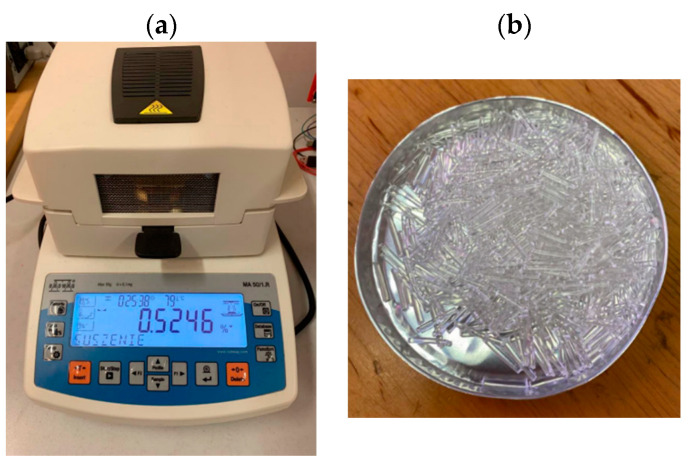
Moisture analyzer (**a**) and filament into pieces for testing moisture content (**b**).

The moisture content of the filament was determined using the weighing method [31] on a Radwag MA 50/1.R moisture analyzer (Figure 3). The measurements consisted of measuring the weight of the samples, drying them, and weighing them again in a dry state. The moisture level was expressed as a percentage as the ratio of the mass of moisture absorbed by the material to the mass of the material after drying.

The samples were produced on a Zortrax M200 Plus printer with a closed chamber using the parameters recommended by the manufacturer and the literature on the subject [8,18,21,28] [Table 1]. Z-SUITE v.2.3 software was used to prepare the control codes.

**Figure 4:**
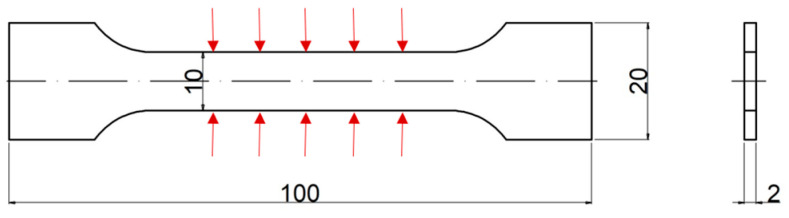
Geometry of samples (dimensions in millimeters) with marked places of cross-section measurement [32].

Measurements (thickness and width) were made for one sample five times at different places in the cross-section. The samples had the shape and dimensions shown in Figure 4.

The mass of the samples was determined with a Radwag WTC 200 electronic scale (RADWAG, Radom, Poland). The thickness and width of the sample were measured with a Mitutoyo Absolute Digimatic 150 mm electronic caliper (Mitutoyo America Corporation, Aurora, IL, USA) and determined as the average of measurements at five points in accordance with Figure 4.

**Figure 5:**
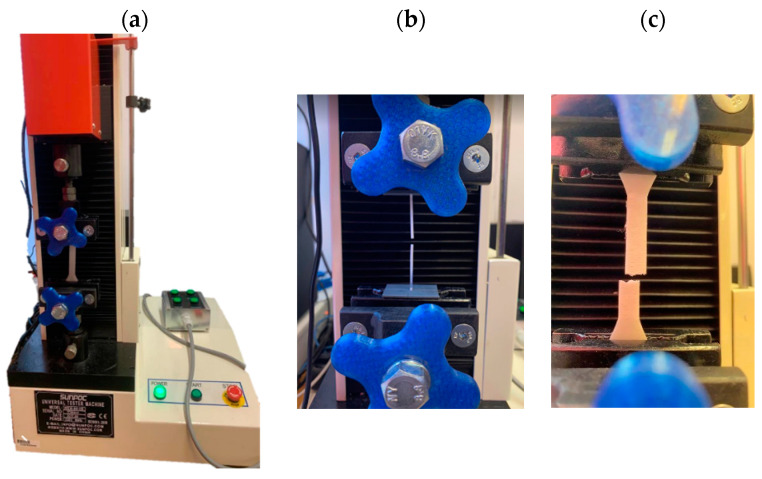
Strength testing machine (**a**) measuring the strength of the filament and (**b**) measuring the strength of the printed samples (**c**).

The tensile strength of the filament and of the printed samples was tested with a SUNPOC WDW-5D-HS testing machine (Figure 5). The quasi-static test was conducted in accordance with ISO 527 [33] at a tensile speed of 2 mm/min.

The surface structure of the samples was also examined. These studies were performed on an INSIZE ISM-PM200SA microscope (INSIZE Co. Ltd., Suzhou, China). It is a compact digital microscope that can record moving and still images. The scope of the magnification range is 10×–200× with a resolution of 1280 × 1024. The assessment of the quality of the surface structure of samples at 180× magnification was studied (uniformity, repeatability, and cross-sectional appearance of the tested samples).

## 3. Results

### 3.1. Dynamics of Moisture Absorption and Desorption

**Figure 6:**
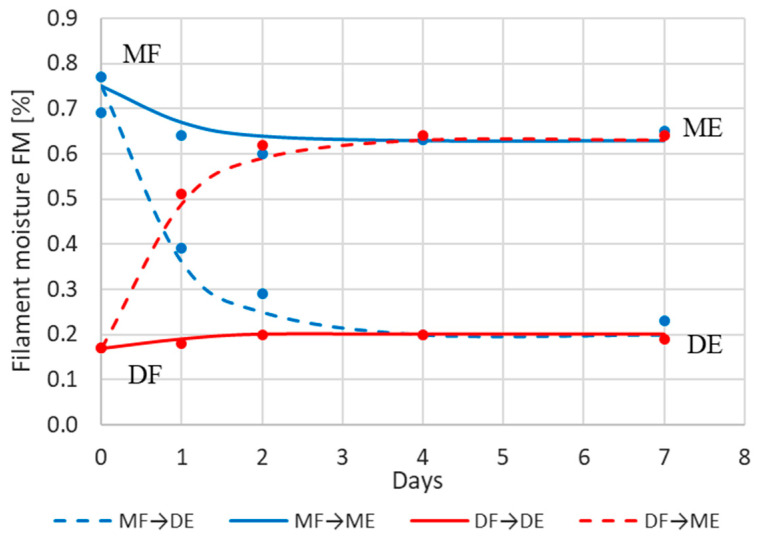
Change in the moisture of dry filament (DF) with an initial moisture of 0.17% and moist filament (MF) with an initial moisture of 0.75% depending on the conditioning time (days) in a climatic chamber in a dry environment (DE) and in a moist environment (ME) [34].

Figure 6 shows the moisture content of the filament after 1, 2, 4, and 7 days of storage in a climatic chamber for the series DF-DE, DF-ME, MF-ME, and MF-DE (see Figure 1).

It is evident that the curves are mirror images of each other; both the absorption and desorption are characterized by an intensity independent of the initial moisture and dependent only on the storage conditions.

These relationships can be described by the following equations:

(a) In the case of absorption,
  $$FM={FM}_{i}+{(FM}_{s}-{FM}_{i})(1-e^{-t/T})$$
(b) In the case of desorption,
  $$FM={FM}_{s}+{(FM}_{i}-{FM}_{s})e^{-t/T}$$

where

- *FM*—filament moisture
- *FM_i_*—initial filament moisture
- *FM_s_*—filament moisture in a stable state
- *t*—time of exposure (conditioning)
- *T*—time constant (in hours or days)

The *FM_i_* and *FM_s_* values depend on external conditions, primarily on the ambient temperature and humidity, as well as on the hygroscopicity of the filament material [29,35,36].

The time constant T depends solely on the hygroscopicity of the material. This is indicated by the results presented in Figure 6. The curves determined for the experimental data have the same value of the time constant T ≈ 20 h (≈0.83 days).

### 3.2. The Influence of Moisture Absorption and Desorption on the Properties of Samples

#### 3.2.1. Tensile Strength

**Figure 7:**
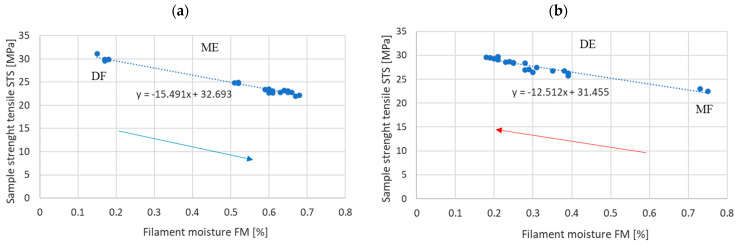
Sample tensile strength (STS) depending on the moisture content of the filament (**a**) for the DF series in ME (absorption) and (**b**) for the MF series in DE (desorption).

The tensile strength of the samples printed from filaments with different moisture content, obtained in the DF–ME and MF–DE series, is shown in Figure 7a,b.

The graphs in Figure 7 show that an increase in filament moisture has a negative impact on the tensile strength of printed samples. In the range of filament moisture from 0.2% to 0.7%, the strength varies from approximately 23 MPa to approximately 30 MPa. These results are consistent with previous studies cited in the first part of this article [8,16,18,37].

However, the strength of this influence does not depend on the history of the filament reaching a given moisture level. For example, for a filament water content of 0.4%, the strength of the samples is approximately 27 MPa, regardless of whether the filament obtained this moisture level as a result of absorption (DF–ME series) or desorption of moisture from the environment (MF–DE series).

The confirmation of this can be seen by comparing the slope coefficients of the linear regression of print strength on filament moisture, as obtained in the DF–ME and MF–DE series (Table 2).

Table 2 shows that the hypothesis about the equality of slope coefficients of the regression lines is rejected at the significance level of *p* > 0.05. This means that both regression lines are significantly similar [38]. In other words, the processes of moisture absorption and desorption do not affect the dependence of the strength of the samples on the moisture content in the material entering the printer.

#### 3.2.2. Dimensional Accuracy

**Figure 8:**
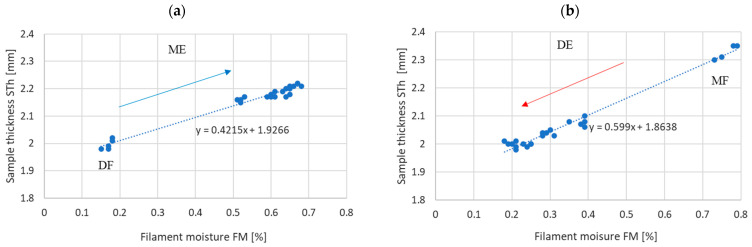
Sample thickness (STh) depending on the filament moisture (FM) obtained (**a**) for the DF series in ME (absorption) and (**b**) for the MF series in DE (desorption).

**Figure 9:**
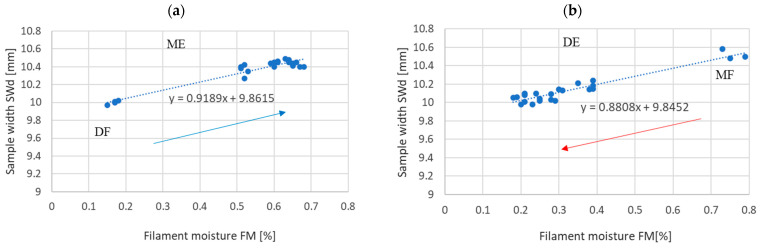
Sample width (SWd) depending on the filament moisture (FM) obtained (**a**) for the DF series in ME (absorption), (**b**) for the MF series in DE (desorption).

Figure 8 and Figure 9 show the dependence of the thickness and width of the samples on the filament moisture content obtained in the DF–ME and MF–DE series.

The results presented in Table 3 indicate that H0’s hypothesis about the equality of the slope coefficients of the regression lines for the thickness and width of the sample—obtained under absorption and desorption conditions, respectively—is rejected at the significance level of *p* > 0.05. This means that the width of the sample and the thickness of the printed sample depend on the moisture content of the material entering the printer, but do not depend on how the moisture is obtained in the material (through absorption or desorption).

#### 3.2.3. Sample Weight

**Figure 10:**
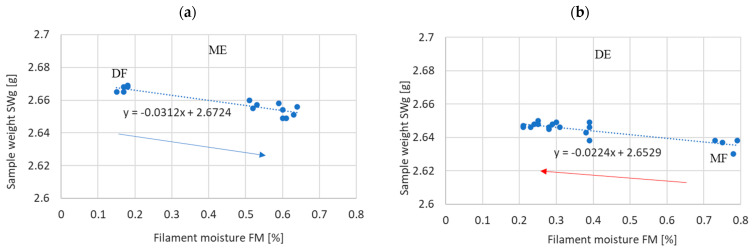
Sample weight (SWg) depending on the filament moisture obtained (**a**) for the series. DF–ME (absorption) (**b**) for the MF–DE (desorption).

The dependence of the sample mass on the moisture content of the filament, including the method of obtaining a given moisture level (by absorption or desorption), is shown in Figure 10.

As with the strength, thickness and width of the print, the strength of this influence does not depend on the history of the filament reaching a given moisture level. This is confirmed by a comparison of the slopes of the regression lines obtained in both cases (Table 4). The statistical test of the equality of the slope coefficients of the regression lines shows that there are grounds to reject the hypothesis of their equality at the significance level of *p* > 0.05.

#### 3.2.4. Surface Structure

**Figure 11:**
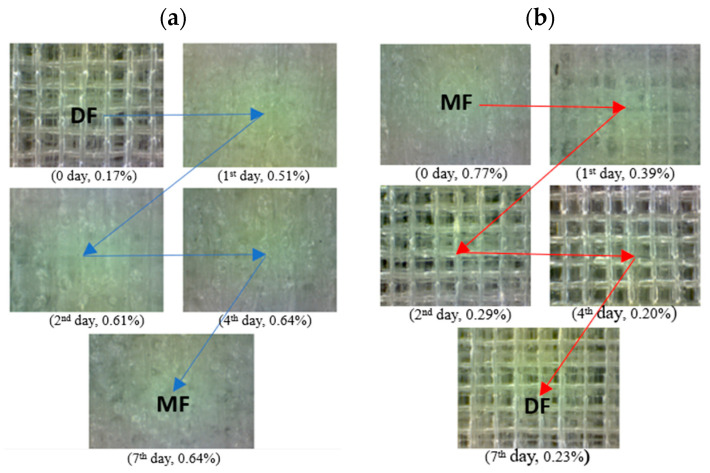
Structure of the sample surface depending on the filament moisture obtained in the series: (**a**) DF–ME (moisture absorption) and (**b**) MF–DE (moisture desorption (180× magnification).

The filament moisture also affects the surface structure of the printed sample (Figure 11).

In the DF–MF series, in prints made from initially dry filament (DF, Figure 11a), a change in structure can be observed after successive days of storage in a humid environment. The surface changes from an anisotropic, regular appearance with clear and well-connected paths to an isotropic structure with indistinguishable material paths. The opposite phenomenon occurs in the MF–DE series, which involves the desorption of moisture from the filament. In the following days (Figure 11b), as the moisture content of the filament decreases, the surface structure of the prints changes from isotropic to anisotropic.

#### 3.2.5. Moisture Absorption by Samples

**Figure 12:**
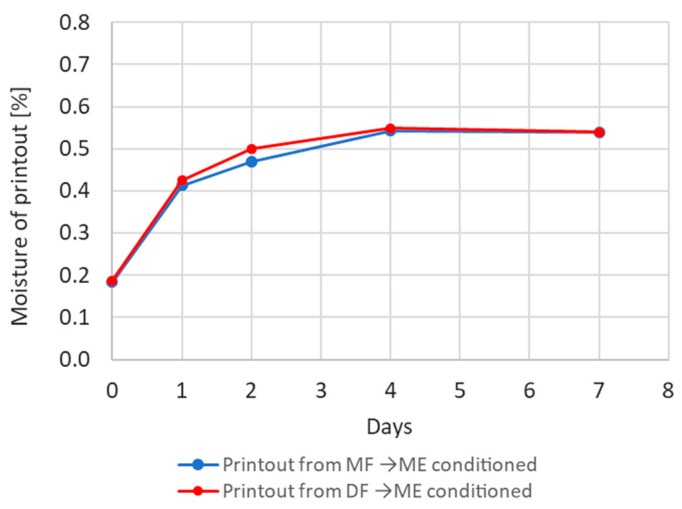
Change in moisture content of samples printed from dry and wet filaments stored in a humid environment.

Figure 12 shows the moisture content of samples (prints) made from moist and dry filaments (DF and MF) and then stored in a climatic chamber in a humid environment—ME (ambient humidity 80% and ambient temperature 30 °C). Moisture content changes in both groups of samples are very similar. The time constant of the equation describing the dynamics of moisture absorption is comparable in both cases (approximately 0.83).

It is characteristic, however, that the printed samples absorb less moisture from the environment than the filament from which they are manufactured. The moisture content of the samples after 4 days, stored in ME conditions, is approximately 0.5% (Figure 12), while the moisture content in the filament stored in the same conditions (ME) is over 0.65% (Figure 6).

**Figure 13:**
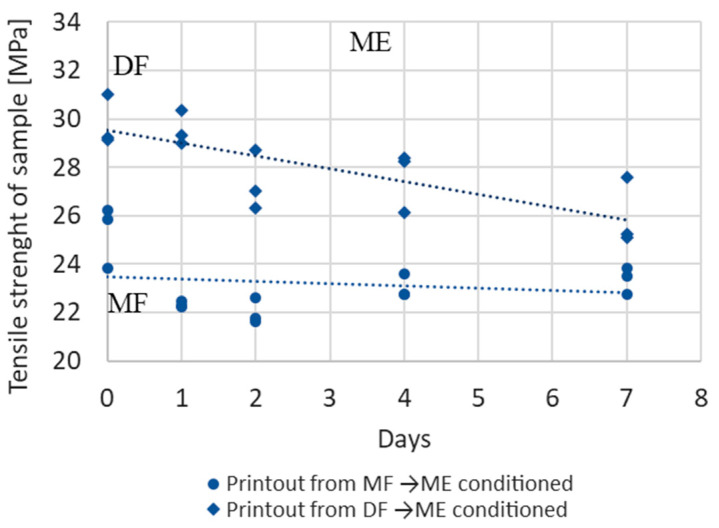
Changes in the strength of samples made from dry (DF) and moist filament (MF) with an initial moisture content of DF = 0.2% and MF = 0.7%, stored in a moist environment (ME) depending on days.

**Figure 14:**
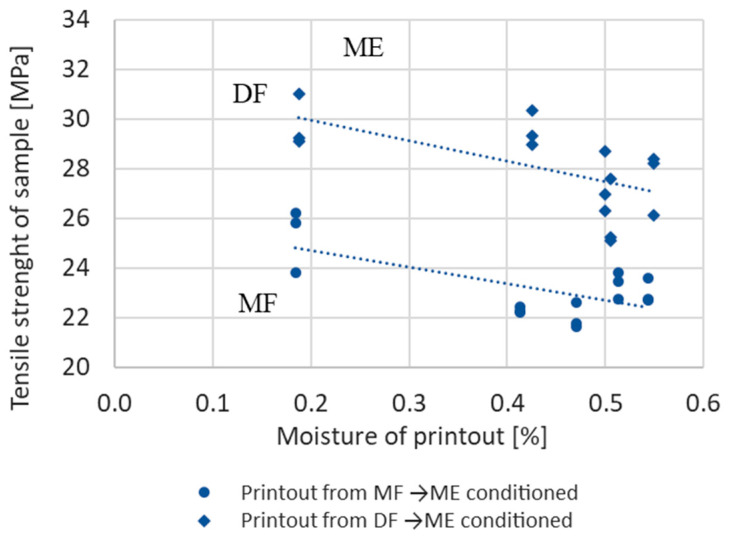
Changes in the strength of samples made from dry (DF) and moist filament (MF) with an initial moisture content of DF = 0.2% and MF = 0.7%, stored in a moist environment (ME) depending on the moisture of printout.

Tensile strength of samples manufactured from dry and moist materials (DF and MF) and then stored in a climatic chamber in a moist environment (ME) is shown in Figure 13 and Figure 14. In Figure 13, the tensile strength is shown depending on the number of days the sample is stored in the chamber, and in Figure 14, the tensile strength is shown depending on the sample moisture.

Regardless of the initial sample moisture, the difference in strength between samples made from dry and moist filament remains the same.

## 4. Conclusions

This research allowed for the formulation of many conclusions, confirming the results published in other works and at the same time showing new aspects of the influence of filament moisture on the properties of printed products.

In particular, the following has been demonstrated:

- The strength of the influence of the filament moisture on the strength of the print (Figure 7), its dimensions (Figure 8 and Figure 9), and surface structure (Figure 11) does not depend on whether the material moisture is the result of absorption or desorption of moisture from the environment. The relationships between the sample strength and size of the sample geometry and the moisture content in the filament material show a linear relationship that is identical regardless of whether the material acquired a given moisture content as a result of absorption or desorption.
- The process of absorption and desorption of moisture into the filament material takes place with intensity depending on the current water content of the filament and the ambient conditions (Figure 6). Regardless of the initial moisture content of the filament, it reaches a level of approximately 0.95% of the level determined after the second day of conditioning.
- An increase in the filament moisture causes a decrease in the tensile strength of the samples (in the range of moisture content from 0.17% to 0.75%, the strength decreases by approximately 25%). The reduction in the strength of the samples is caused by changes leading to the weakening of the structure of the sample.
- Bubbles are formed in the structure of a sample made from moist material, which is the result of a high concentration of vapors from volatile substances resulting from the evaporation of moisture contained in the filament. In samples manufactured from dry filament, there are no bubbles in the material and the material is evenly distributed.
- The moisture contained in the filament causes the sample to swell. Figure 8 and Figure 9 show that the thickness of a sample made from moist filament is about 6% greater than the thickness of a sample made from dry filament, and the width is correspondingly greater by about 10%. Moreover, in the process of printing samples from moist filament, the base material is lost along with escaping gases. This is indirectly confirmed by the results in Figure 10, i.e., that the weight of a sample that uses moist filament is approximately 1.5% lower than the weight of a sample that uses dry filament. Both phenomena—material swelling and material loss—make the structure of a print made from a moist filament looser than that of a sample made from a wet filament, which is a direct cause of the reduction in tensile strength of the sample.
- Printed samples absorb less moisture from the environment than the fiber from which they are made (before inserting the filament into the printer). The time constant of the moisture absorption curve is comparable in both cases. This phenomenon is probably caused by the fact that filament absorbs moisture deeply, while in the case of printed samples, absorption occurs only on the surface.
- The tensile strength of samples manufactured from both dry and moist filament exposed to moisture leads to a decrease in strength. This decline is comparable in both cases.

In future studies, it is advisable to investigate the effect of changes in filament moisture content under natural storage conditions on the stability of the printing process. The following should also be considered: different filament suppliers, FDM/FFF equipment with different designs, e.g., material feed system, and heating of the working chamber. It is also advisable to extend the range of process parameters.

Another research topic worthy of attention is the depth of moisture absorption in ABS materials. This applies not only to the filament, but also to the FDM-manufactured product. Currently, it is evident that in the case of FDM, the effect of moisture in the processed material is transferred to the entire volume of the manufactured product. A product made of moist material has a porous structure, which renders it more susceptible to moisture penetration into the product.

## Figures and Tables

**Table 1:** Printing parameters.

Parameter	Value
Layer thickness	0.19 mm
Nozzle temperature	275 °C
Printing speed	30 mm/s
Degree of filling	100%
Filling pattern	grid, 45 grad
Orientation of samples on the table	horizontal
Nozzle diameter	0.4 mm
Table temperature	80 °C.

**Table 2:** Regression line and correlation coefficients of filament moisture and sample tensile strength (STS).

	Regression Line	Correlation Coefficient	Hypothesis of Equality of Regression Line Slope	
			t-Statistics	The *p*-Value of Rejecting the Hypothesis
Moisture absorption	STS = −15.49 FM + 32.69	r = −0.993	−1.954004	*p* = 0.0599
Moisture desorption	STS = −12.51 FM + 31.46	r = −0.934		

**Table 3:** Regression equations and correlation coefficients of filament moisture and sample thickness (STh) and sample width (SWd).

	Regression Line	Correlation Coefficient	Hypothesis of Equality of Regression Line Slope	
			t-Statistics	The *p*-Value of Rejecting the Hypothesis
Moisture absorption	STh = 0.422 FM + 1.927	r = −0.934	1.931603	*p* = 0.0568
Moisture desorption	STh = 0.599 FM + 1.863	r = −0.993		
Moisture absorption	SWd = 0.919 FM + 19.862	r = 0.945	−0.362022	*p* = 0.719
Moisture desorption	SWd = 0.881 FM + 9.845	r = 0.970		

**Table 4:** Regression equations and correlation coefficients of filament moisture and sample weight (SWg).

	Regression Line	Correlation Coefficient	Hypothesis of Equality of Regression Line Slope	
			t-Statistics	The *p*-Value of Rejecting the Hypothesis
Moisture absorption	SWg = −0.0312 FM + 2.6724	r = −0.719	1.944347	*p* = 0.0591
Moisture desorption	SWg = −0.0224 FM + 2.6529	r = −0.414		

## Data Availability

Data are contained within the article.
